# Myopia progression varies with age and severity of myopia

**DOI:** 10.1371/journal.pone.0241759

**Published:** 2020-11-20

**Authors:** Pavan Kumar Verkicharla, Priyanka Kammari, Anthony Vipin Das

**Affiliations:** 1 Myopia Research Lab, Prof. Brien Holden Eye Research Centre, LV Prasad Eye Institute, Hyderabad, Telangana, India; 2 Brien Holden Institute of Optometry and Vision Sciences, LV Prasad Eye Institute, Hyderabad, Telangana, India; 3 Department of eyeSmart EMR & AEye, LV Prasad Eye Institute, Hyderabad, Telangana, India; 4 Department of Comprehensive Ophthalmology, LV Prasad Eye Institute, Hyderabad, Telangana, India; National Eye Institute, UNITED STATES

## Abstract

**Objective:**

To investigate annual myopia progression in individuals from South Indian states across different age groups, and its association with age of onset and severity of myopia.

**Methods:**

This retrospective study included the data of 6984 myopes (range: 1–30 years), who visited at least twice to LV Prasad Eye Institute and on whom a standard retinoscopy technique was performed to determine refractive error. Based on spherical equivalent (SE) refractive error, individuals were classified into mild, moderate, high and severe myopic groups. Myopia progression was calculated as difference between SE at 1-year follow-up visit and at baseline. To determine the age-specific myopia progression, individuals were further categorized as myopes who are at least 15 years or younger and those who are above 15.

**Results:**

The mean annual progression of myopia was influenced by both the age group (p < 0.001) and severity type of myopia (p < 0.001). The overall mean myopia progression ranged from -0.07 ± 0.02 D (standard error) to -0.51 ± 0.02 D across different age groups with maximum change in refractive error noted in children aged 6–10 years and the least in adults aged 26–30 years. Myopia progression was greater in severe myopes, followed by high, moderate, mild myopes and in individuals aged ≤ 15 years compared to those aged >15 years (-0.45 ± 0.01 vs. 0.14 ± 0.01, p < 0.001). Severe myopes alone had similar annual myopia progression rate irrespective of age (i.e ≤15 and >15 years, p = 0.71). Early onset of myopia was associated with high myopia in adulthood.

**Conclusion:**

The magnitude of myopia progression in children from South Indian states is comparable to that of Caucasians and Chinese. The greater progression in ‘severe myopes’ across different age groups emphasize the need for regular follow-ups, monitoring axial lengths, and anti-myopia strategies to control myopia progression irrespective of the age and degree of myopia.

## Introduction

Myopia is one of the common refractive errors in children, worldwide. It is estimated that about one billion myopes globally are at risk of developing myopia related complications by year 2050 [1, 2]. The prevalence and progression of myopia are known to vary with various factors such as age, age of onset of myopia, the severity of myopia, country, and ethnicity [3, 4]. Previous studies indicated that the mean annual myopia progression in children was about half-a-diopter in Europeans (-0.55D) and a slightly higher progression rate in Asians (-0.82D) [5]. The Northern Indian Myopia study that involved 10000 school children aged 5 to 15 years from one state of India (Delhi) reported an annual myopia progression of -0.27 ± 0.42 D [6]. A recent study that involved Indian children and young adults based on a hospital-based data indicated that 4.3% of the myopes had pathologic myopia similar to that of Caucasians and East Asians [7, 8].

There is a lacuna in the literature about myopia progression in Indians, especially the refractive error changes in different age groups and with the severity of myopia. Given the potential role of ethnicity and geographic location on the progression of myopia, information on the pattern of progression of myopic refractive error across different age groups in Indian children could help clinicians in choosing appropriate anti myopia treatment strategies. In that front, this study aimed to estimate the annual myopia progression in Indians and its relationship with age, the age of onset, and severity of myopia.

## Methods

This retrospective study was conducted at the L V Prasad Eye Institute (LVPEI), Hyderabad, India. The required data of individuals who visited at least twice to any of the LVPEI centers located in India from January 2010 to January 2016 for ophthalmic consultation was extracted from the electronic medical records (EMR) database of LVPEI. This study was approved by the Institutional Review Board of the LVPEI, Hyderabad (Approval Number: LEC 02-18-043) and was conducted in accordance with the Tenets of the declaration of Helsinki. All individuals who came for eye examination had signed on the written informed general consent prior to their clinical examination approving the use of their data for research purposes. In the case of a child (< 18 years), the parents/guardian of the child provided the consent.

**Fig 1:**
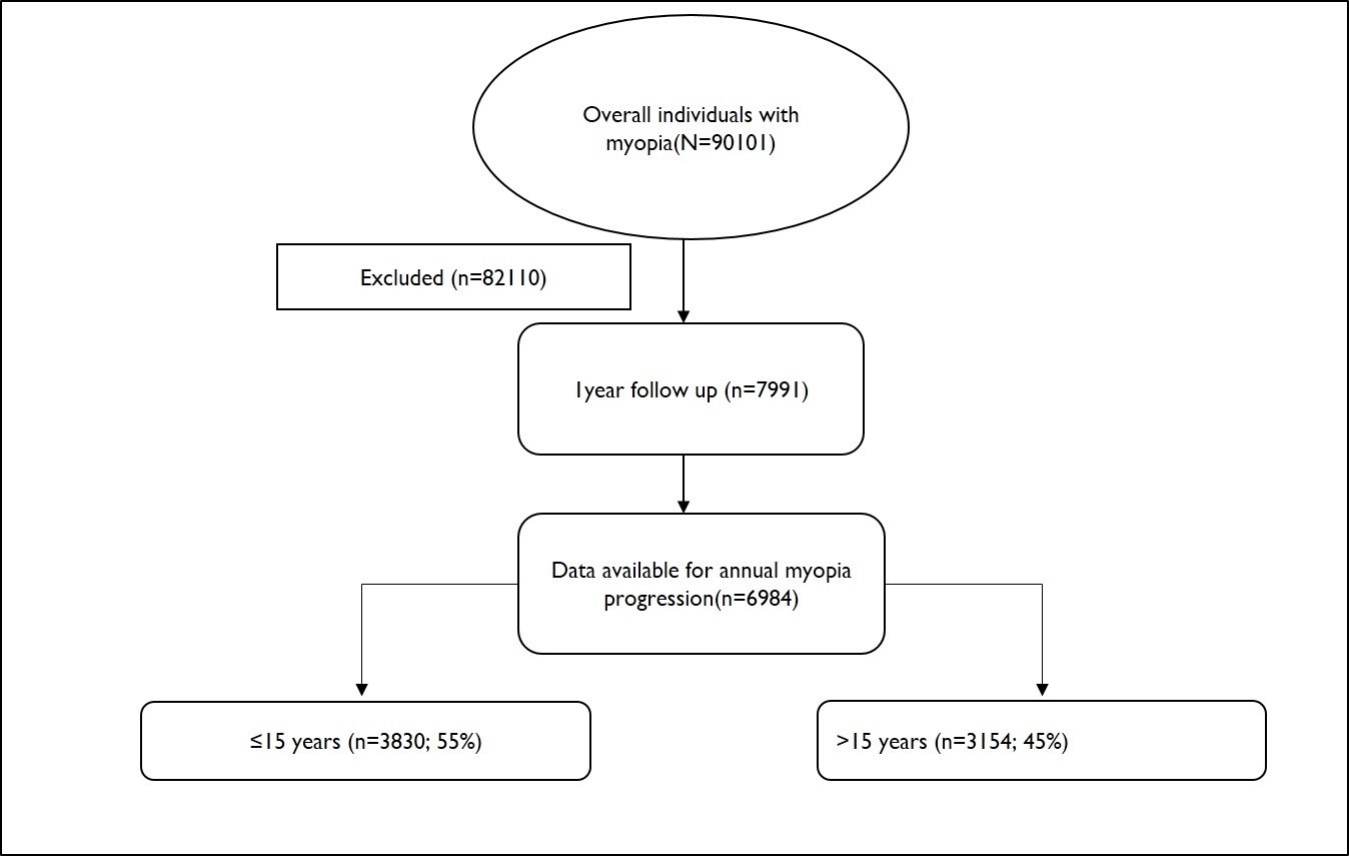
Flowchart with sample distribution based on inclusion and exclusion criteria.

Individuals aged 1 to 30 years and only with the diagnosis of ‘myopic refractive error’ in their first visit (taken as a baseline) were included in the study. The medical records of individuals with any missing data of required variables and presence of any other ocular condition/pathology such as pterygium, post-operative refractive surgeries, corneal disorders, cataract, etc. that influences the refractive error was not considered for analyses. Overall, there were 6984 (7.7%) myopes who met the inclusion criteria and whose refractive error data was available for estimating myopia progression for one year of a total 90101 myopic individuals who visited LVPEI (Fig 1). The follow-up duration of each participant was determined based on the number of days between each visit (i.e. 335 to 390 day’s gap between each visit was considered for one-year progression). The collected data included variables such as age, the age of onset of myopia, gender, demographic details and refractive error (sphere, cylinder and axis). Refraction of the eye was recorded based on standard objective hand-held retinoscopy technique performed by an Optometrist while the individual viewed at the non-accommodative target placed at 4 meters from eye. According to the protocol followed at the LV Prasad Eye institute from where the data was collected, cycloplegic refraction was performed usually in children younger than 16 years to determine the refractive error. The cycloplegic refraction was performed in 21% of total included myopes and in 40% of children younger than 16 years. The spherical equivalent refractive error was defined as the sum of the spherical and half of the cylindrical power. Myopia was defined as spherical equivalent refraction (objective) worse than –0.5 diopters (D). Based on degree of myopia at baseline, individuals were categorized as mild (≤-0.50D to -3.00D), moderate (<-3.00D to -6.00D), high myopia (<-6.00D to -9.00D) and severe myopia (<-9.00D). To investigate how the progression of myopia in individuals with extreme high myopia (without any ocular pathology) vary with that of regular high myopia, we have defined myopia as severe myopia too in this study. Astigmatism was defined based on the cylindrical component of overall refraction of eye (≥ -0.25 D) and was further classified as with-the-rule(WTR) if the axis lied between15 degrees on either side of the horizontal meridian (N = 3167; 68%), against-the-rule(ATR) if the axis was between 15 degrees on either side of the vertical meridian (N = 1280; 27%) and oblique astigmatism (OA) if the axis was from 15 to 75 degrees or 105 to 165degrees (N = 245; 5%).

Myopia progression was calculated as the difference between spherical equivalent refractive error at 1-year follow-up visit and at baseline. Further analysis was performed only on right eyes data as the spherical equivalent refraction was not significantly different between right and left eyes (*p = 0*.*74*). Considering the influence of age, emmetropization and plasticity of eye on change in refractive error, especially in children, all the myopes were categorized into 6 groups in 5-year intervals i.e. 1–5, 6–10, 11–15, 16–20, 21–25, 26–30 years. Our data analysis also showed agreement to such a bimodal distribution of the progression of myopia based on age and also corroborates with the previous studies that indicated slowing of the progression of myopia around age 15 years [9]. As a decent number of previous studies reported the progression of myopia in individuals aged up to 15 years, the participants in this study was further divided into two major divisions (myopes who are at least 15 years or younger and those who are above 15) to determine age-specific myopia progression for comparisons with previous studies.

To investigate the association of “apparent age of onset of myopia” on the development of high myopia in adulthood, information related to the age of onset of myopia was analyzed only for adults aged 20 years or above. Age cutoff of 20 years was chosen to ensure that we could subgroup into 5 year age bins for easy comparisons. Overall, the information related to age of onset of myopia in adults aged greater than 20 years was available in 1682 individuals (who either also belonged to 1 year progression data or might have just come only once after the age of 20 years). Based on information obtained from the history section of the medical records, “history of wearing glasses since “x” years” was equated to the age of onset of myopia. For the participants who did not wear any refractive correction, prior to the consultation at LVPEI or were not diagnosed to have myopia earlier to the visit at LVPEI, their age of onset of myopia was considered at that particular visit date.

Statistical analysis was performed using Microsoft Excel (version 2016) along with IBM SPSS statistical software (version 20). Considering that the data was obtained from four tertiary eyecare centers of LVPEI located in Andhra Pradesh (two centers), Telangana and Orissa, further sub-analysis was performed to see if any difference in outcome exists due to regional variations.

Majority of the individuals belongs to three major states, namely Andhra Pradesh (N = 3567; 51%), Telangana (N = 1843; 27%) and Orissa (N = 1289; 19%). The remaining of the individuals belong to other states of India (N = 285; 4%). Two-tailed unpaired t-tests were performed to investigate the association of gender and age of onset of myopia. Multiple ANOVA tests were performed separately to find the association of myopia progression with age, the age of onset of myopia, type of astigmatism: WTR, ATR, and OA; location of the participants: Telangana, Andhra Pradesh and Orissa).

## Results

Of all the myopic individuals included in this study, 95% (N = 6645) of the individuals had iso-metropic refractive error and only 5% had aniso-metropic refractive error (≥1.00 D difference between two eyes). Table 1 shows the baseline spherical equivalent and annual myopia progression values based on age, gender and severity of myopia. Mean age and spherical equivalent refraction of the included myopes was 15.7 ± 6.5 years (standard deviation) and -3.24 ± 3.03 D, respectively. There were more males (n = 3723; 53%) compared to females (n = 3261; 47%) and a number of individuals with a mild degree of myopia (65%) compared to that of moderate (23%), high (7%) and severe myopia (5%).

**Table 1:** Baseline mean spherical equivalent values and myopia progression based on age, gender and severity of myopia.

	N	Baseline SE	Annual progression
Total	6984	-3.24 ± 3.03	-0.33 ± 0.68
**Age**			
0–5	308	-3.81 ± 3.94	-0.49 ± 0.86
6–10	1231	-3.31 ± 3.30	-0.51 ± 0.79
11–15	2291	-3.13 ± 2.61	-0.46 ± 0.68
16–20	1466	-3.46 ± 3.10	-0.23 ± 0.56
21–25	1032	-3.19 ± 3.16	-0.11 ± 0.51
26–30	656	-2.86 ± 2.90	-0.07 ± 0.54
**Age groups**			
≤15 years	3830	-3.24 ± 2.97	(-0.45 ± 0.01
>15 years	3154	-3.25 ± 3.09	0.14 ± 0.01
**Gender**			
Males	3723	-3.21 ± 3.06	-0.31 ± 0.66
Females	3261	-3.29 ± 2.99	-0.36 ± 0.70
**Severity of myopia**			
Mild	4541	-1.65 ± 0.69	-0.29 ± 0.60
Moderate	1604	-4.33 ± 0.84	-0.36 ± 0.69
High	475	-7.36 ± 1.01	-0.40 ± 0.81
Severe	364	-13.04 ± 3.32	-0.67 ± 1.10

**Fig 2:**
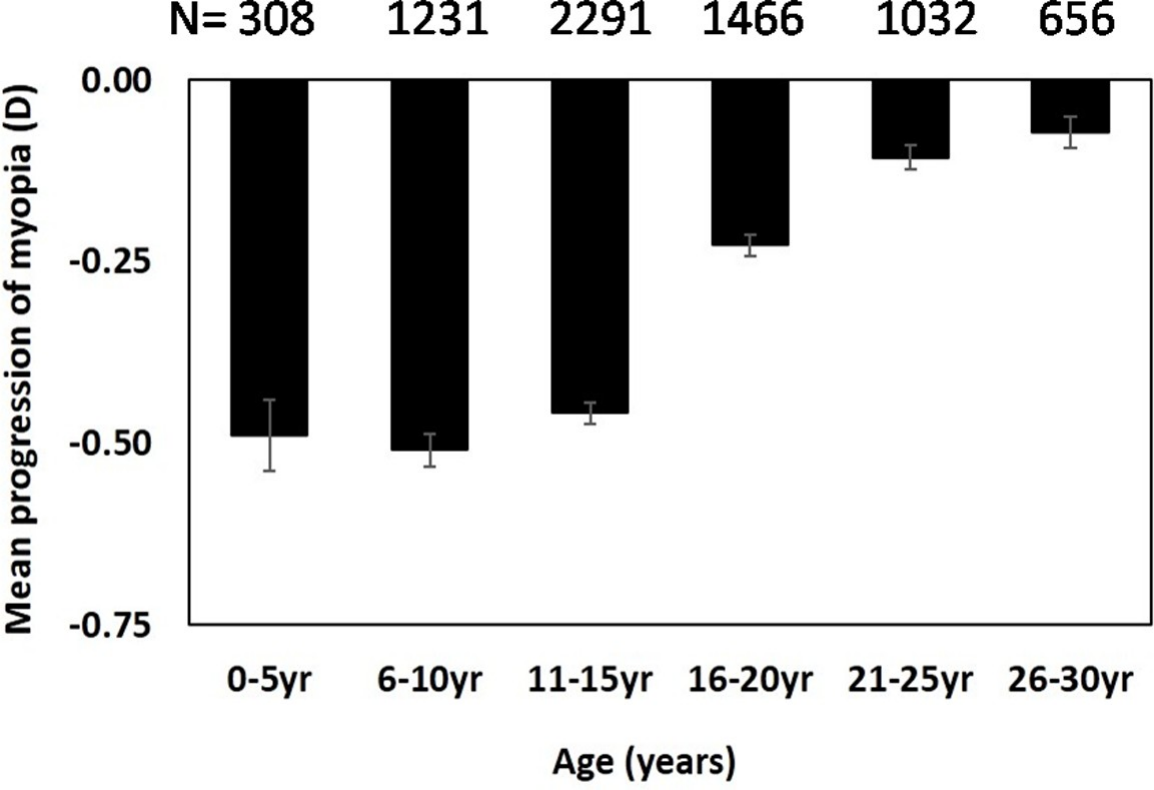
Mean myopia progression based on age. Error bars represent standard error of the mean.

**Fig 3:**
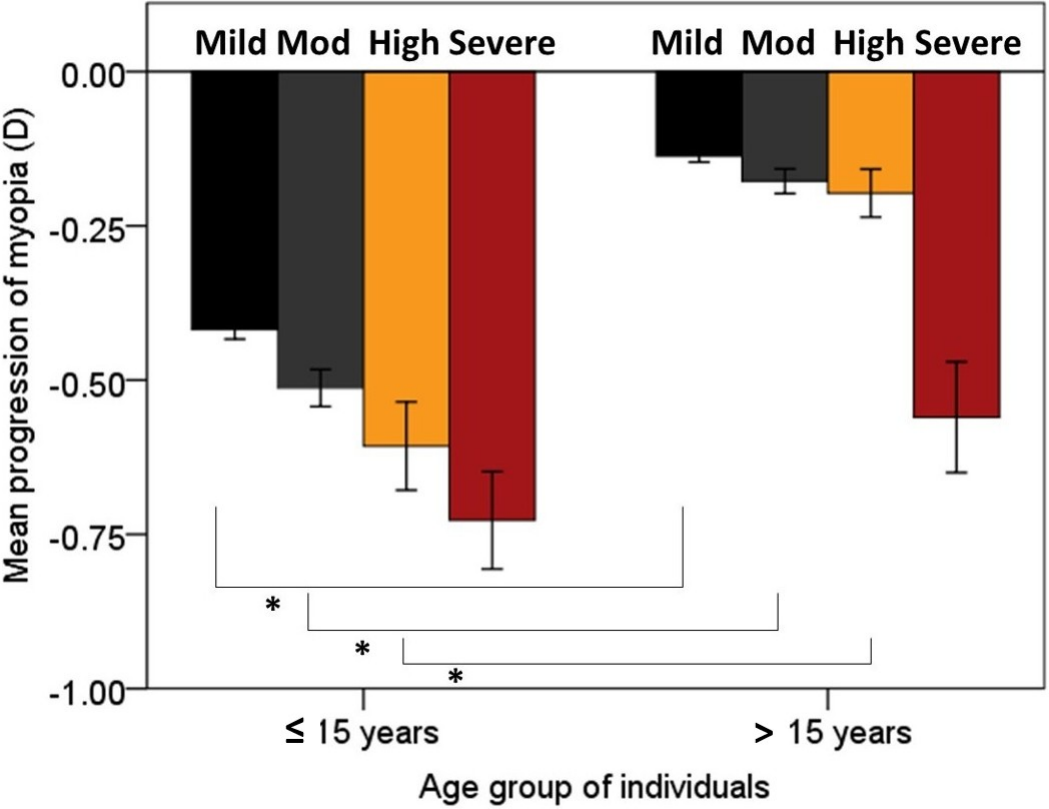
Mean annual progression of myopia based on the severity of myopia in myopes younger than 15 years and those 15 years or older. Error bars represent standard error of the mean. Note (*) indicates the significance of variables.

The mean annual progression of myopia was influenced by both the age group (ANOVA, *F* (5,6983) = 76.23, p < 0.001) and severity type of myopia (ANOVA, *F* (3,6983) = 36.08, p < 0.001). Post-hoc analysis indicated a bimodal distribution of the progression of myopia based on age with the progression rates was not significantly different between age groups 1–5, 6–10, and 11–15 years and between 16–20, 21–25 and 26–30 years. The overall mean myopia progression ranged from -0.07 ± 0.02 D (standard error) to -0.51 ± 0.02 D across different age groups with maximum annual change in refractive error noted in children aged 6–10 years and the least in adults aged 26–30 years (Fig 2). Further analysis based on stratification of broader age group indicated significantly greater myopia progression in individuals aged ≤ 15 years compared to those aged >15 years (-0.45 ± 0.01 vs. 0.14 ± 0.01, p < 0.001). Myopia progression was greatest in severe myopes, followed by high, moderate, mild myopes (Table 1). Post-hoc analysis indicated significant inter-group differences for severity group (p<0.01 for all expect for mild vs moderate myopes, p = 0.11). Eighteen percent of myopes aged ≤15 years and seven percent of myopes aged >15 years had rapid progression of myopia (>1D). Myopia progression in severe myopic individuals alone was similar in both the age groups (i.e ≤15 and >15 years) and was not statistically significant (p = 0.71) (Fig 3).

Both gender (males: -0.31 ± 0.01 D vs. females: -0.36 ± 0.01 D, p = 0.09) and the location of individuals did not influence the overall progression of myopia (ANOVA, *F* (2, 6696) = 32.85, p = 0.07). The mean annual myopia progression in individuals from Andhra Pradesh, Telangana and Orissa are -0.3 ± 0.01D, -0.30 ± 0.02 D, and -0.37 ± 0.02 D, respectively. Astigmatism was found in 67% (n = 4692) of the individuals. Mean myopia progression was not significantly different among individuals with different types of astigmatism (ANOVA, *F* (2,4689) = 2.63, p = 0.72); corresponding mean myopia progression values are: with-the-rule (-0.31 ± 0.01 D), against-the-rule (-0.26 ± 0.19 D), oblique astigmatism (-0.32 ± 0.04 D).

**Fig 4:**
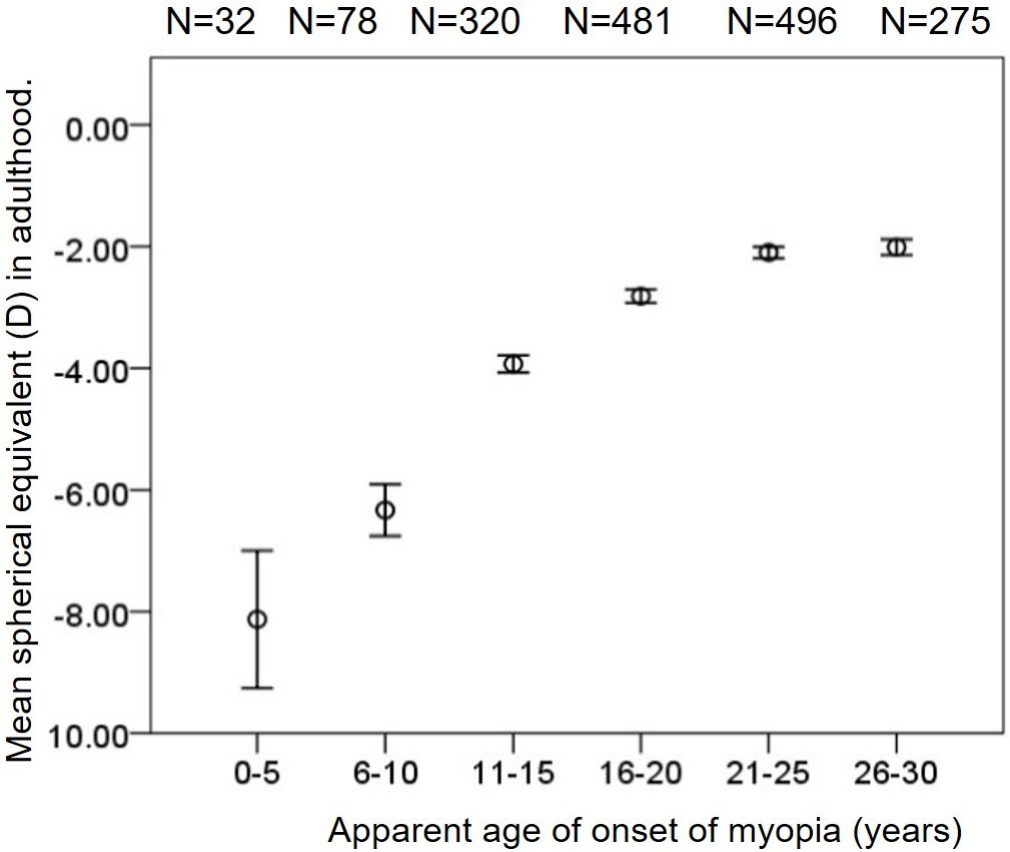
Mean spherical equivalent based on the age of onset of myopia, only in adult individuals who are at least 20 years and above.

The mean spherical equivalent refraction in adults who developed myopia at an early age had significant high myopia compared to the ones who developed at later age, i.e. in 0–5 years and 6–10 years the mean spherical equivalent was -8.13 ± 1.13 D and -6.33 ± 0.43 D respectively, and in individuals with 21–25 years and 26-30years of age the mean spherical equivalent was-2.10 ± 0.09 D and -2.01 ± 0.13D, respectively (p<0.001) (Fig 4).

## Discussion

The results from this study indicate that the annual myopia progression in children and young adults from South Indian states varied with age, the age of onset of myopia and severity of myopia, but not with gender, geographic location of the individual and type of astigmatism. The mean myopia progression was about half-a-diopter in individuals aged ≤15 years of age (-0.47 to -0. 51D) and less than a quarter of diopter in individuals aged above 15 years. Similar differences in the magnitude of progression between the age groups were reported in Chinese individuals aged 7 to 70 years [10, 11].

The annual progression values are similar to those in Caucasian children (aged 6 to 15 years) living in Australia (-0.31 to -0.41D), Europe (-0.55D), UK and USA (-0.34 D to -0.50D) and in East Asian countries like China and Singapore (-0.31 to -1.2 D) [5, 12–14]. The results from this study indicate a greater mean progression than that of the findings from the Northern Indian Myopia Study performed on urban school children (-0.27 ± 0.42 D) [6]. In this data set, participants were majorly from three different states of South India and it is possible that these regions are in the similar level of urbanization and due to which the myopia progression was similar between these groups. Similar results were reported by others in Chinese and Caucasian children [15, 16]. The reason for the variations in the myopia progression among different countries and within India (North and South Indian states) could possibly be explained by the variations in the location, lifestyle and ethnicity among different population groups. [20] How lifestyle varies within the locations of India is beyond the scope of this manuscript. Individuals with a higher degree of myopia had greater myopia progression compared to those with a mild degree of myopia. A similar association was reported too in Taiwanese, Chinese and Singaporean school children [3, 17–19]. The exact mechanism on why high and severe myopes progress at a faster rate compared to that of mild myopes is not clear, an indirect relation can be speculated towards an association with the earlier age of onset of myopia. The finding that there was similar mean annual myopia progression rates in severe myopia group irrespective of the age is reported for the first time. It is possible that the severe myopia cohort might have a different causal relationship with myopiogenesis unlike physiological myopia and could be due to the greater influence of genes in myopiogenesis and progression in high myopia [20, 21]. However, given that there was no information about parental myopia in the data set, the interpretation about the relationship between the potential role of genes on the greater progression rate in severe myopes should be made with caution. The early onset of myopia was associated with high myopia in adulthood compared to the ones who developed at a later age. Although there are subtle differences in the way “early onset of myopia” was defined among various research studies, the results from this study corroborate with that of the previous studies in that the earlier the age of onset of myopia might impose a greater risk of developing high myopia in adulthood [3, 16].

The biggest strength of the current study is the inclusion of a big data for determining the annual myopia progression estimates, i.e. 6984 individuals. This is the largest data used thus far to determine myopia progression in the Indian population from South Indian states, including the participants from three different geographic locations of India like Andhra Pradesh, Telangana and Orissa. This study does have some limitations. Firstly, the retrospective nature of the data set may lead to inter-observer variability in the assessment of refraction at each follow-up visit and could have caused the greater variability in the outcome. Our study is a hospital-based study where a sizeable number of patients walks in only if they have some vision-related problem. However, considering that a strict criterion was used for exclusion of participant with other ocular condition/pathology that influences the refractive error, caution on the generalizability of the results of this study could be decreased. It is possible that children with high refractive error during early childhood (specially aged 1–5 years) might visit the tertiary eye care center which could have led to mean values shift towards the myopic side in this specific age group. Other limitations were the unavailability of various potential factors such as parental myopia, time spent outdoors, the actual age of onset of myopia and thus explanation of the role of various risk factors attributing to myopia progression (specially the severe myopes) in Indian population is beyond the scope of this retrospective study. The location from where the participant came was obtained only from the postal address that was given for the communication purposes at the time of registration and thus may not be a representative of the location at which they grew up or living. Further studies involving separate data from rural and urban areas, with data related to various factors such as exposure to light levels/time outdoors, are required to provide insights on myopia progression pertinent to the specific ethnic scenario.

In conclusion, the magnitude of myopia progression in Indian children from the South Indian states are comparable to that of Caucasians and Chinese, and warrants the implementation of various strategies for myopia control in Indians. This finding of the greater progression in ‘severe myopes’ compared to that of the mild myopes across different age groups emphasize the need for regular follow-ups with short intervals, monitoring axial lengths, and application of anti-myopia strategies to control myopia progression irrespective of the age and severity of myopia.

## Supporting information

S1 Data (ZIP)

## References

[1] Holden B. A. et al Nearly 1 billion myopes at risk of myopia‐related sight‐threatening conditions by 2050–time to act now. Clin Exp Optom 98, 491–493,(2015). 10.1111/cxo.12339 26769175

[2] Resnikoff S. et al Myopia–A21stCenturyPublicHealthIssueMyopia–A21stCenturyPublic Health Issue. Invest Ophthalmol Vis Sci 60, Mi–Mii,(2019). 10.1167/iovs.18-25983 30817824 PMC6396683

[3] Chua S. Y. et al Age of onset of myopia predicts risk of high myopia in later childhood in myopic Singapore children. Ophthalmic Physiol Opt 36, 388–394,(2016). 10.1111/opo.12305 27350183

[4] Dolgin E. The myopia boom. Nature 519, 276–278,(2015). 10.1038/519276a 25788077

[5] Donovan L. et al Myopia progression rates in urban children wearing single-vision spectacles. Optometry and Vision Science 89, 27–32,(2012). 10.1097/OPX.0b013e3182357f79 21983120 PMC3249020

[6] Saxena R. et al Prevalence of myopia and its risk factors in urban school children in Delhi: the North India Myopia Study (NIM Study). PLoS ONE 10, e0117349–e0117349,(2015). 10.1371/journal.pone.0117349 25719391 PMC4342249

[7] Dhakal R., Goud A., Narayanan R. & Verkicharla P. K. Patterns of posterior ocular complications in myopic eyes of Indian population. Sci Rep 8, 13700,(2018). 10.1038/s41598-018-29536-x 30209314 PMC6135820

[8] Verkicharla P. K., Ohno-Matsui K. & Saw S. M. Current and predicted demographics of high myopia and anup date of its associated pathological changes. OphthalmicPhysiolOpt 35,465–475, (2015). 10.1111/opo.12238 26303444

[9] Sheeladevi S. et al Prevalence of refractive errors in children in India: a systematic review. Clinical and Experimental Optometry 101, 495–503,(2018). 10.1111/cxo.12689 29682791

[10] Chen Y. et al Methodology of the ZOC-BHVI High Myopia Cohort Study: The Onset and Progression of Myopic Pathologies and Associated Risk Factors in Highly MyopicChinese. Ophthalmic Epidemiol 25, 31–38,(2018). 10.1080/09286586.2017.1338733 28891727

[11] Sun Y. Y. et al Effect of uncorrection versus full correction on myopia progression in12-year-old children. Graefes Arch Clin Exp Ophthalmol 255, 189–195,(2017). 10.1007/s00417-016-3529-1 27796670

[12] Saw S. M. et al Incidence and progression of myopia in Singaporean school children. Invest Ophthalmol Vis Sci 46, 51–57,(2005). 10.1167/iovs.04-0565 15623754

[13] Fan D. S. P. et al Prevalence, incidence, and progression of myopia of school children in Hong Kong. Investigative Ophthalmology and Visual Science 45, 1071–1075,(2004). 10.1167/iovs.03-1151 15037570

[14] French A. N., Morgan I. G., Burlutsky G., Mitchell P. & Rose K. A. Prevalenceand5-to6- year incidence and progression of myopia and hyperopia in Australian school children. Ophthalmology 120, 1482 1491,(2013). 10.1016/j.ophtha.2012.12.018 23522969

[15] Ma Y. et al Different patterns of myopia prevalence and progression between internal migrant and local resident school children in Shanghai, China: a 2-year cohort study. BMC Ophthalmology 18, 53–53, (2018). 10.1186/s12886-018-0716-3 29471798 PMC5824479

[16] Hyman L. et al Relationship of age, sex, and ethnicity with myopia progression and axial elongation in the correction of myopia evaluation trial. Arch Ophthalmol 123, 977–987, (2005). 10.1001/archopht.123.7.977 16009841

[17] Zhou W. J. et al Five-YearProgressionofRefractiveErrorsandIncidenceofMyopiainSchool-Aged Children in Western China. J Epidemiol 26, 386–395, (2016). 10.2188/jea.JE20140258 26875599 PMC4919484

[18] Lam C. S., Edwards M Millodot M. & Goh W.S. A2-yearlongitudinalstudyofmyopia progression and optical component changes among Hong Kong schoolchildren. Optom Vis Sci 76, 370–380, (1999). 10.1097/00006324-199906000-00016 10416931

[19] Hsu C. C. et al Risk factors for myopia progression in second-grade primary school children in Taipei: a population-based cohort study. Br J Ophthalmol 101, 1611–1617, (2017). 10.1136/bjophthalmol-2016-309299 28315834

[20] Young T. L. Molecular genetics of human myopia: an update. Optom Vis Sci 86, E8–e22, (2009). 10.1097/OPX.0b013e3181940655 19104467 PMC3718050

[21] Tedja M. S. et al IMI–Myopia Genetics Report IMI–Myopia Genetics Report. Invest Ophthalmol Vis Sci 60, M89–M105, (2019). 10.1167/iovs.18-25965 30817828 PMC6892384

